# chewBBACA: A complete suite for gene-by-gene schema creation and strain identification

**DOI:** 10.1099/mgen.0.000166

**Published:** 2018-03-15

**Authors:** Mickael Silva, Miguel P. Machado, Diogo N. Silva, Mirko Rossi, Jacob Moran-Gilad, Sergio Santos, Mario Ramirez, João André Carriço

**Affiliations:** ^1^​ Instituto de Microbiologia, Instituto de Medicina Molecular, Faculdade de Medicina, Universidade de Lisboa, Lisbon, Portugal; ^2^​ Department of Food Hygiene and Environmental Health, Faculty of Veterinary Medicine, University of Helsinki, Helsinki, Finland; ^3^​ School of Public Health, Faculty of Health Sciences, Ben-Gurion University of the Negev, Beer Sheva, Israel; ^4^​ Public Health Services, Ministry of Health, Jerusalem, Israel

**Keywords:** chewBBACA, multilocus sequence typing, schema, gene-by-gene, allele calling

## Abstract

Gene-by-gene approaches are becoming increasingly popular in bacterial genomic epidemiology and outbreak detection. However, there is a lack of open-source scalable software for schema definition and allele calling for these methodologies. The chewBBACA suite was designed to assist users in the creation and evaluation of novel whole-genome or core-genome gene-by-gene typing schemas and subsequent allele calling in bacterial strains of interest. chewBBACA performs the schema creation and allele calls on complete or draft genomes resulting from *de novo* assemblers. The chewBBACA software uses Python 3.4 or higher and can run on a laptop or in high performance clusters making it useful for both small laboratories and large reference centers. ChewBBACA is available at https://github.com/B-UMMI/chewBBACA.

## Data Summary

1. Assembled genomes used for the tutorial were downloaded from NCBI in August 2016 by selecting those submitted as *Streptococcus agalactiae* taxon or sub-taxa. All the assemblies have been deposited as a zip file in FigShare (https://figshare.com/s/9cbe1d422805db54cd52), where a file with the original ftp link for each NCBI directory is also available.

2. Code for the chewBBACA suite is available at https://github.com/B-UMMI/chewBBACA while the tutorial example is found at https://github.com/B-UMMI/chewBBACA_tutorial.

**Impact Statement**

The chewBBACA software offers a computational solution for the creation, evaluation and use of whole genome (wg) and core genome (cg) multilocus sequence typing (MLST) schemas. It allows researchers to develop wg/cgMLST schemes for any bacterial species from a set of genomes of interest. The alleles identified by chewBBACA correspond to potential coding sequences, possibly offering insights into the correspondence between the genetic variability identified and phenotypic variability. The software performs allele calling in a matter of seconds to minutes per strain on a laptop but is easily scalable for the analysis of large datasets of hundreds of thousands of strains using multiprocessing options. The chewBBACA software thus provides an efficient and freely available open source solution for gene-by-gene methods. Moreover, the ability to perform these tasks locally is desirable when the submission of raw data to a central repository or web services is hindered by data protection policies or ethical or legal concerns.

## Introduction

Read mapping approaches using single nucleotide polymorphisms (SNP)/single nucleotide variants (SNV) have been widely used for studying bacterial genomes [1]. However, gene-by-gene (GbG) approaches have also been advocated in the context of genomic epidemiology as an expansion of multilocus sequence typing (MLST) [2] allowing portability, scalability and independence from a defined reference strain. For these reasons, GbG has increasingly gained popularity and has been adopted by PulseNet International as the method for bacterial strain discrimination using high-throughput sequencing [3]. GbG relies on comparing the draft genome of a strain of interest against a pre-defined schema, typically using an approach based on blast [4]. This schema can be composed of core loci, which are present in all or the great majority (e.g. 95 %) of the analysed strains (core genome MLST schemas or cgMLST), or including all loci detected in the strains of interest. The latter are referred to as whole-genome or pan-genome MLST schemas (wgMLST or pgMLST).

A locus in a schema can be a complete coding sequence (CDS) or a subsequence of it, as in traditional MLST. Defining a locus as a CDS allows linking of the variability found to potential changes in proteins and thus phenotype. The definition of locus is currently dependent on the algorithm used for comparing loci and defining the schema, hampering comparison between different GbG approaches.

Only a few software packages are available for GbG allele calling and no tools are available for schema creation and validation. Two commercial platforms offer GbG analyses: Ridom SeqSphere+ (http://ridom.de/seqsphere/) and Bionumerics (http://www.applied-maths.com/applications/wgmlst). Since these are proprietary, closed-source software, their GbG allele-calling algorithms are incompletely described [5, 6], although Ridom schemas have been publicly released (http://www.cgmlst.org/).

BIGSdb was the first open-source freely available platform allowing cgMLST analysis [7] and is currently the basis of the PubMLST website (https://pubmlst.org/). More recently, EnteroBase has provided comprehensive cgMLST and wgMLST schemas and an allele calling engine for three major food-borne bacterial pathogens (https://enterobase.warwick.ac.uk/). A limitation of EnteroBase is the requirement to submit reads to the website or to public repositories (NCBI SRA/EBI ENA), since currently no stand-alone versions of their allele calling algorithm are available. At present, the only published open-source stand-alone GbG allele-calling algorithm from assemblies is Genome Profiler [8] which, however, uses a single CPU core, making it unsuitable for large-scale analyses. Recently, MentaLiST, a software to perform allele calling directly from reads has also been presented [9], but it relies on existing schemas and allele definitions.

We developed chewBBACA to be a complete stand-alone pipeline for GbG analyses, including constructing and validating novel cg/wgMLST schemas and performing CDS allele calling suitable for large scale studies. chewBBACA performs the schema creation and allele calls on complete or draft genomes resulting from *de novo* assemblers.

## Theory and implementation

**Fig. 1.**
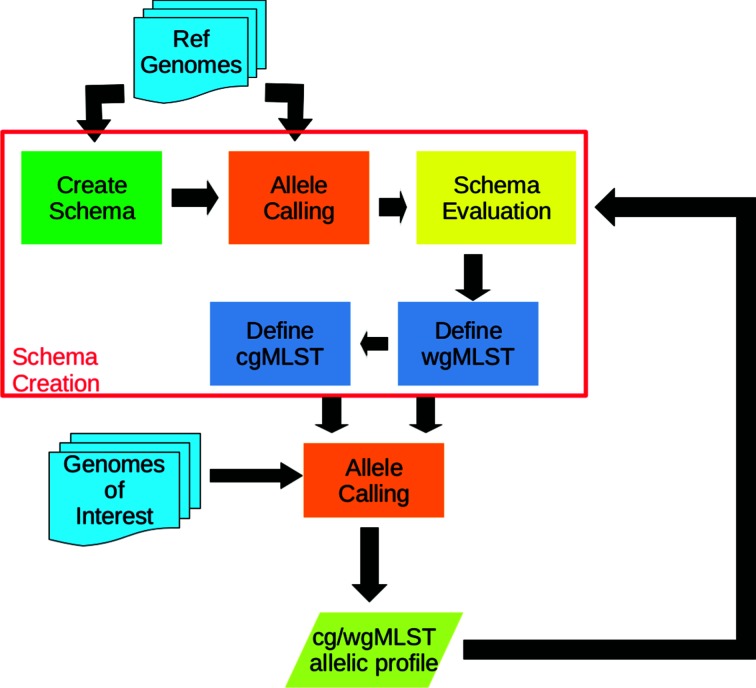
chewBBACA workflow from schema definition to schema evaluation

chewBBACA addresses the three main concepts necessary for GbG: schema creation, allele calling and schema evaluation. A general workflow of such processes is presented in Fig. 1.

### wg/cgMLST schema creation

ChewBBACA allows the definition of wg/cgMLST schemas from user-provided complete genomes or draft assemblies, focusing on excluding paralogous loci, detection of contaminated/poor-quality assemblies and supporting user decisions towards the identification of the most appropriate schema through interactive graphic data analysis.

To achieve this goal several steps are needed which involve different operations within chewBBACA.

The first step is the *CreateSchema* operation that offers an iterative approach for CDS comparison in the selection of loci, which is more computationally efficient than using software such as OrthoMCL [10] or CD-hit [11]. To create a wgMLST schema, the user provides a set of genomes in FASTA format. The algorithm first defines the CDSs of each genome using Prodigal [12]. In the next step, all the CDSs in the genomes are compared in a pairwise fashion, resulting in a single FASTA file containing all CDSs identified in the genomes after a two-step evaluation process. Firstly, all the CDSs having identical sequences to other CDSs but being smaller in length are removed and the larger CDS is kept. At the same time, the algorithm also removes all CDSs with a length less than indicated in the ‘−l’ parameter. In the second step, the remaining CDSs are clustered in unique loci by performing an all-against-all blastp search and calculating the blast score ratio (BSR) [13]. CDSs with a BSR pairwise comparison equal or greater than 0.6 are considered alleles of the same locus and the larger allele (in bp) is kept in the list. This procedure defines the schema as a set of CDSs, each representing the largest single allele of distinct loci.

The second step is to perform the allele call using the resulting set of loci. ChewBBACA’s *AlleleCall* operation is then used to populate the schema with alleles using the same genomes used for its creation. This step allows the identification and exclusion of possibly paralogous loci. The allele-calling algorithm detects if a CDS in the genome under analysis matches more than one locus in the schema, indicating that those loci could be paralogous, and outputs a list of such loci to be removed from the wgMLST schema using the *RemoveGenes* operation or to be further investigated. From the created wgMLST schema, cgMLST schemas can be defined by selecting the loci that are present in a predetermined percentage of the analysed strains, typically 95–99 %, by the use of the *TestGenomeQuality* operation.

### Allele calling algorithm

**Fig. 2.**
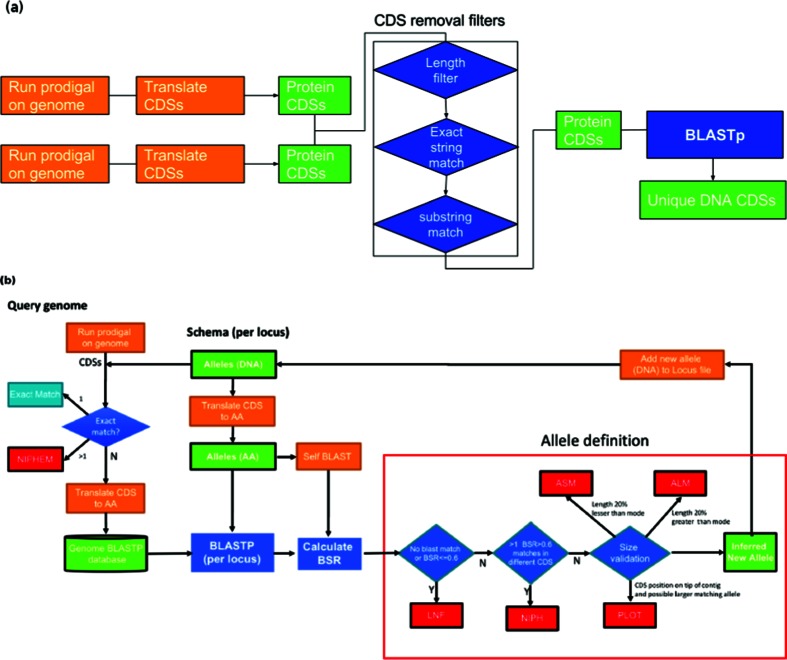
(a) chewBBACA pairwise comparison for schema creation algorithm (b) chewBBACA allele calling algorithm.

**Fig. 3.**
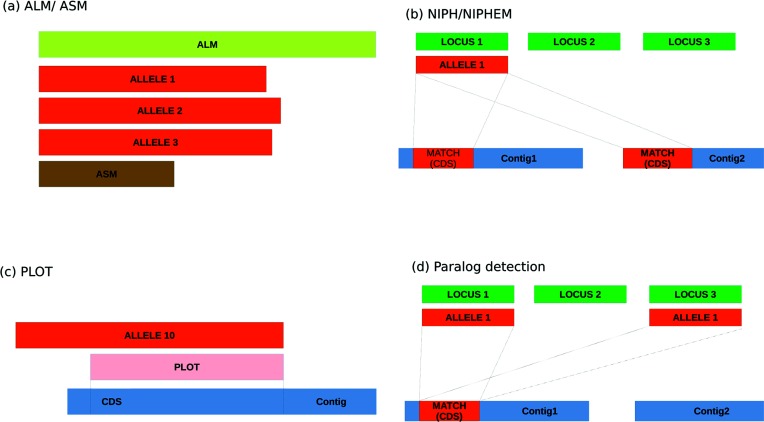
chewBBACA allele definition outputs. (a) Size exclusion of alleles 20 % smaller or larger than the allele length mode for the loci (b) Detection of loci duplication on the draft genome (c) Detection of locus identified on the 5′ or 3′ ends of the contig (d) Detection of paralogous loci

The *AlleleCall* operation is based on CDSs identified by Prodigal [12]. Firstly, the CDSs with 100 % nucleotide identity to alleles present in the database are classified. All remaining CDSs are compared using a blastp BSR approach, allowing the detection of alleles with divergent DNA sequences but similar encoded proteins. This allows the identification of alleles that would be considered absent loci with blastn, while retaining the full diversity found at the DNA sequence level. The algorithm is defined as presented in Fig. 2. A blastp database is created, containing all the translated CDSs identified by Prodigal in the query genome. A 100 % DNA identity comparison is performed on all the genome of interest CDSs against each locus allele database. If an exact match is found, an allele identification is attributed to the CDS (and tagged as *EXC – Exact Match*, in the statistics output). If not, a blast BSR approach is used to identify the allele. To improve computational efficiency, chewBBACA performs the similarity search on each locus in the schema separately, performing the jobs in parallel using the specified number of CPUs. For each locus, a short list containing the most divergent alleles is queried against the BLASTP database. The BSR is calculated for each hit and based on these results and a size validation step, the locus is either considered not found (tagged as *LNF – Locus Not Found*) or a new allele of the locus is inferred. The size validation step excludes alleles larger than or smaller than 20 % of the locus allele length mode (Defined as *ASM – Alleles Smaller than Mode* or *ALM – Alleles Larger than Mode*) (Fig. 3a). Furthermore, the identification of loci as duplicated in the genome of interest is also reported. Such matches are identified as *Non-Informative Paralogous Hits* (*NIPH*), if at least two CDSs have best matches with alleles of the same locus but presenting less than 100 % identity, or *NIPHEM – NIPH Exact Match* if 100 % identity to existing alleles is detected (Fig. 3b). Furthermore, the algorithm detects whether the CDS match is close to the 5′ or 3′ ends of a contig and a larger allele that contains the matched sequence would exceed the contig length. Such sequences are tagged as *Possible Locus On the Tip* (*PLOT*) (Fig. 3c). Finally, the *AlleleCall* operation identifies possible paralogous sequences (as described above) checking if there are CDS matching alleles in two or more different loci (Fig. 3d). After running each genome, the loci database is updated with the newly found alleles and, whenever required, a locus short-list is also updated with a new divergent allele.

### Schema evaluation

The operation *SchemaEvaluator* allows the assessment of the suitability of including each locus in a schema through a suite of functions to graphically explore and evaluate the type and extent of allelic variation detected in each of the chosen loci. This operation also creates multiple sequence alignments of the alleles of each locus using MAFFT [14] and reconstructs neighbour-joining trees using ClustalW2 [15], allowing the exploration of the potential consequences of the variability at each locus. This operation can be used to analyse any existing cg/wgMLST schema, including those created by other methodologies, since the analysis input is a set of FASTA files, one per locus, with all identified alleles.

A more complete description of each operation and their functionalities is available at https://github.com/B-UMMI/chewBBACA/wiki

### Benchmark

**Fig. 4.**
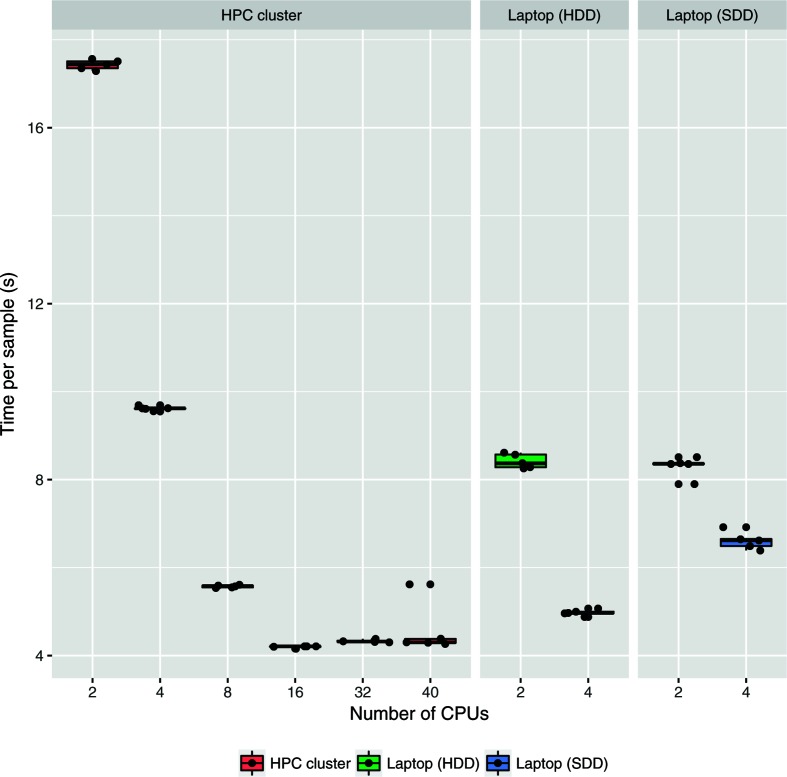
Benchmarking of chewBBACA's allele-calling algorithm for bacterial genome assemblies (approximately 2 Mb) using a cgMLST schema of 1264 loci on a HPC cluster and two laptops with different storage devices. The allele calling was executed five times for each CPU data point.

The performance of chewBBACA's allele-calling algorithm was evaluated for *Streptococcus agalactiae* assemblies (an approximately 2 Mb genome) using a cgMLST schema of 1264 loci. Benchmarks were performed on a high-performance cluster (HPC) with Intel Xeon E5-2630 v4 @ 2.20 GHz CPUs, up to 256 Gb RAM and an SSD distributed storage in RAID1; a laptop with Intel Core i5-7200U @ 2.50 GHz×4 CPUs, 8 Gb RAM and a NVMe SSD storage; and a laptop with Intel Core i7-3630QM @ 2.40GHz×8 CPUs, 8 Gb RAM and a SATA2 HDD storage. Allele calling was conducted for 100 *S. agalactiae* assemblies using the HPC cluster and the two laptops (Fig. 4). Each CPU data point was run five times. In terms of CPU performance, the time it took to run each sample decreased almost linearly up to 32 CPUs, at which point disk, possibly I/O storage, access becomes the bottleneck and no increase in performance is observed. At peak performance using 32 CPUs in the HPC, allele calling takes approximately 2.5 s for each sample in the benchmark dataset.

### Usage example

A tutorial providing a complete usage example, demonstrating the creation of a schema for *Streptococcus agalactiae*, from publicly available complete genomes and assemblies available at NCBI/ENA is provided at https://github.com/B-UMMI/chewBBACA_tutorial.

## Conclusion

The chewBBACA suite was developed to allow performance of GbG analyses in high-end Unix-based laptops but chewBBACA can also be easily run in HPC, facilitating its adoption into large-scale automated analysis pipelines. The good performance of the software on current laptops and in HPCs, allows flexible implementation in small laboratories or large reference centres. The allele-calling engine of chewBBACA uses FASTA files with draft assemblies or complete genomes as input and returns as output an allelic profile matrix and a set of FASTA files containing the full allelic diversity of each locus. Currently available cg/wgMLST schemas, can be adapted to run using chewBBACA’s allele-calling engine. The chewBBACA suite is the first, to our knowledge, to provide schema creation tools and to enforce CDS allele calling, which can be important for evaluating phenotypic diversity, including the identification of the potential mechanisms underlying the success of particular clones. Since there is an urgent need for bioinformatics solutions that will allow the development of nomenclature-based schemas [16], future work will focus on centralized repositories for schemas and allele definitions that can be synchronised with local allele-calling outputs to facilitate the development of common schemas and nomenclatures for cg/wgMLST, allowing a more widespread application of GbG methodologies in public health.

## Data bibliography

1. Assembled genomes used for the tutorial were downloaded from NCBI in August 2016 by selecting those submitted as *Streptococcus agalactiae* taxon or sub-taxa. All the assemblies have been deposited as a zip file in FigShare (https://figshare.com/s/9cbe1d422805db54cd52), where a file with the original ftp link for each NCBI directory is also available.
2. Code for the chewBBACA suite is available at https://github.com/B-UMMI/chewBBACA while the tutorial example is found at https://github.com/B-UMMI/chewBBACA_tutorial.
